# Pattern of hydromorphone use in King Abdulaziz Medical City-Central Region (KAMC-CR)

**DOI:** 10.1038/s41598-021-88276-7

**Published:** 2021-04-22

**Authors:** Saja Alhabardi, Hind Almodaimegh, Maha Alammari

**Affiliations:** 1 Saudi Food and Drug Authority, Riyadh, Saudi Arabia; 2 King Abdulaziz Medical City, Riyadh, Saudi Arabia

**Keywords:** Palliative care, Respiratory distress syndrome

## Abstract

Hydromorphone is a semi-synthetic opioid that acts mainly on the μ-opioid receptor. Hydromorphone has a fast onset of action, usually within 5 min, and its effectiveness peaks at approximately 20 min, which makes it favourable in the postoperative setting. It plays a role in the management of moderate to severe chronic pain. The most common adverse effects of hydromorphone are hypotension, bradycardia, and respiratory distress. The aim of this study was to determine the trend in the use of hydromorphone analgesics and to evaluate hydromorphone-related toxicity in King Abdulaziz Medical City-Central Region **(**KAMC-CR). A retrospective, cross-sectional study was carried out in KAMC-CR, and medical and pharmacological data were retrieved from electronic health records for adult patients who used hydromorphone between December 2014 and December 2015. The characteristics of the enrolled patients, including measured blood pressure, heart rate, respiration rate, oxygen saturation, and pain severity score, were collected. Moreover, we identified patients who received naloxone as a hydromorphone antidote. A total of 153 patients were included; 64.1% were male and 35.8% were female. The mean age of the included patients was 55.5 years old (+/− 18.6). Although the majority of patients reported an improvement in pain severity, 75 patients (49%) needed naloxone to overcome adverse effects of hydromorphone. The mean age of patients who received naloxone was 56.2 years old (+/− 20.5), their mean weight was 75.9 kg (+/− 17.2), and 61.3% of them were male (n = 46). Among those who received naloxone, 84% patients (n = 63) had received hydromorphone intravenously. The risk of respiratory depression was significantly higher in patients who received hydromorphone intravenously (IV) than in those who received it orally (*p* = 0.02). Hydromorphone can have adverse effect. Thus, we recommend evaluating cardiac parameters, oxygen saturation, respiration rate, and pain severity before administering hydromorphone, particularly in patients who have a high risk of cardiorespiratory adverse events, such as patients with cardiac disease, asthma, or chronic obstructive pulmonary disease. Additionally, we recommend the use of appropriate hydromorphone doses in cases of conversion from other opioid therapy or changes between oral and IV routes of the administration of hydromorphone. Moreover, we recommend establishing a policy to restrict the prescription of hydromorphone to avoid the overuse of hydromorphone and minimize the risk of adverse effects and medication errors.

## Introduction

Opioid analgesics have long been used to treat pain in cancer patients. The indications for opioids were expanded to include acute and chronic non‐cancer pain. As a consequence of extending these indications, opioid utilization has increased significantly, and the global opioid market was valued at USD 25.4 billion in 2018 and is projected to expand by 1.8% annually^1–3^.

A recent systemic review included 38 studies that assessed the rates of opioid misuse, abuse, and addiction in patients with chronic pain. The prevalence of opioid misuse averaged between 21 and 29%. Furthermore, the rates of addiction averaged between 8 and 12%. The prevalence of opioid abuse was reported in only one study, with averages rates of 0.08% and 0.81%^4^.

The rates in Saudi Arabia are lower than those reported internationally. In a cross-sectional study carried out in a chronic pain clinic in the King Faisal Specialist Hospital and Research Centre in 2019, the prevalences of opioid misuse, abuse and dependence in chronic pain patients attending chronic pain clinics were 12.8%, 9.1% and 3.2%, respectively^5^.

Hydromorphone is a semi-synthetic opioid that acts mainly on μ-opioid receptors. Hydromorphone has a fast onset of action, usually within 5 min, and its effectiveness peaks at approximately 20 min, which makes it favourable in the postoperative setting. Moreover, hydromorphone is five times as potent as morphine when administered by the oral route and 8.5 times as potent when administered by the IV route^6^.

The safety and tolerability of hydromorphone is addressed in the product information on the label. The most common serious adverse events associated with hydromorphone are life-threatening respiratory depression in patients with chronic pulmonary disease and severe hypotension, adrenal insufficiency, coma, elevated intracranial pressure, seizure, suicidal thoughts, apnoea, drug dependence, and drug withdrawal syndrome in elderly, cachectic, or debilitated patients. Moreover, dosing errors have been reported for the oral hydromorphone solution due to confusion between mg and mL, which have resulted in accidental overdoses and deaths^7^.

Bradycardia and orthostatic hypotension are the most commonly reported adverse effects of hydromorphone and are reported in up to 20% of patients using hydromorphone in the post-operative setting. This is due to peripheral arteriolar and venous dilation. Hydromorphone minimally affects the cerebral circulation except when the partial pressure of carbon dioxide (PaCO_2_) increases as a result of respiratory depression, and the effect is dose dependent, particularly for hydromorphone administered via the epidural route^6^.

Other opioids, such as morphine, oxycodone, fentanyl, and methadone, all cause a dose-dependent decrease in respiration, with apnoea occurring at high doses. Additionally, morphine, hydromorphone, hydrocodone, and meperidine can lead to histamine release and, as a result, can cause significant decreases in systemic vascular resistance and blood pressure. In a systemic review that searched six databases for randomized controlled trials comparing patient-controlled analgesia (PCA) with ketamine plus morphine/hydromorphone and PCA with morphine/hydromorphone for post-operative pain in adult patients, respiratory depression as an adverse event was reported in the ketamine plus morphine/hydromorphone group in 12 trials (1030 patients; RR, 0.59; 95% CI, 0.30 to 1.17), and cardiovascular adverse effects, including arrhythmia, hypotension, hypertension, or bradycardia, were reported in two trials (120 patients; RR, 1.51; 95% CI, 0.14 to 16.28)^8,9^.

Currently, there have been no studies on the patterns and consequences of hydromorphone utilization in Saudi Arabia, including respiratory and cardiovascular adverse events. This information is important because although hydromorphone and morphine are equally effective, hydromorphone is more expensive^10,11^.

## Methods

A retrospective, cross-sectional study was carried out in the KAMC-CR. Medical and pharmacological data were retrieved from the electronic health records for adult patients who used hydromorphone for post-operative or chronic pain between December 2014 and December 2015. Patients were excluded if they were placed on concomitant opioid therapy, if they were receiving medications that are well known to cause respiratory disease or if they had respiratory or cardiac diseases. The cardiac adverse events related to hydromorphone that were considered were bradycardia and hypotension. Bradycardia was defined as a heart rate less than 60 beats per minute (HR < 60 b/m)^12^, and hypotension was defined as a reduction in systolic blood pressure (SBP) to less than 90 mmHg or by ≥ 20 mmHg or a reduction in mean arterial pressure (MAP) to less than 65 mmHg or by ≥ 10 mmHg^13^. Respiratory adverse events related to hydromorphone were defined as a reduction in the respiration rate (RR) to less than 12 breaths/min or a reduction in oxygen saturation (SpO_2_) to less than 90%^14^. Pain intensity was assessed with the Verbal Rating Scale (VRS) over several days^15^. The route of administration, dose, and dosing frequency of hydromorphone and demographic and clinical data of patients who received naloxone were collected. The vital signs for eligible patients were followed every day if the patients were hospitalized patients or during clinic visits if the patients were outpatients. All methods/experiments were carried out in accordance with the relevant guidelines and regulations (Declaration of Helsinki), and informed consent was obtained from all subjects.

### Statistical analyses

Statistical analyses were performed with SPSS 19.0 software (IBM, NY, USA). Categorical data are expressed as percentages and frequencies and were analysed with the chi-square test. Descriptive statistics for the continuous data are expressed as the means ± standard deviations (SDs) and were compared by Student’s t-tests. All statistical assessments were 2-tailed, and the level of significance was set at *p* = 0.05.

### Declarations

All methods/experiments were carried out in accordance with the relevant guidelines and regulations (Declaration of Helsinki).

### Ethics approval

The ethics committee of King Abdullah International Medical Research Center approved the study.

### Informed consent

Informed consent was obtained from all subjects.

## Results

A total of 153 patients were included; 64.1% were male (n = 98) and 35.9% were female (n = 55). The mean age of the included patients was 55.5 years old (+/− 18.6), and their mean body weight was 83.54 kg (+/− 15.2). Cardiac adverse events were reported in 47.7% of the patients (n = 73). Of them, 23.53% of the patients experienced bradycardia (n = 36), and 24.2% of patients developed hypotension (n = 37). Respiratory adverse events were reported in 26.8% of patients (n = 41); among them, 21.6% of the patients experienced respiratory depression (n = 33), while only 5.23% of the patients developed oxygen desaturation (n = 8) (Table 1).

**Table 1:** Demographic data and adverse events in the subjects who received hydromorphone.

Number of patients (N)	153
Age (years)	Mean = 55.5
**Sex**	
Female	55
Male	98
Weight (kg)	83.54
Cardiac adverse events	(n = 73)
Bradycardia	36
Hypotension	37
Respiratory adverse events	(n = 41)
Respiratory depression	33
Oxygen desaturation	8
**Pain assessment**	**Mean**
No pain	81
Mild	29
Moderate	33
Severe	10

Although the majority of patients reported an improvement in pain severity, 49% (n = 75) required 0.4 to 2 mg IV naloxone repeated every 2 to 3 min to overcome the adverse effects of hydromorphone on the patients’ cardiac and respiratory functioning (Table 2). The mean age of the patients who received naloxone was 56.2 years old (+/− 20.5), and their mean weight was 75.9 kg (+/− 17.2). Approximately 61.3% of the patients who required naloxone were male (n = 46). However, there was no significant difference in naloxone use between males and females. Among the patients who received naloxone, 36 patients reported no pain, 13 reported mild pain, 20 reported moderate pain, and only 6 patients reported severe, uncontrolled pain. In total, 84% of the patients (n = 63) received hydromorphone intravenously, and the risk of respiratory adverse events was significantly higher in those who received hydromorphone intravenously than in those who received it orally or epidurally (*p* = 0.02) (Table 3).

**Table 2:** Characteristics of subjects who received naloxone therapy.

Number of patients (N)	75
Age (years)	Mean = 56.2
**Sex**	
Female	29
Male	46
*p* value	*p* = 0.51
Weight (kg)	Mean = 75.9
**Pain assessment**	**Mean**
No pain	36
Mild	13
Moderate	20
Severe	6
**Route of administration**	
Intravenous (IV)*	63
**Oral**	9
Epidural/PCA**	3
	N = 75
**Hydromorphone dose**	2
6 mg	3
4 mg	10
2 mg	4
1 mg	13
0.4 mg	20
0.3 mg	20
0.2 mg	3
0.1 mg	*p* < 0.05

*Intravenous route.

**Patient-controlled analgesia.

**Table 3:** Relationship between route of administration and hydromorphone-related cardiac adverse events and respiratory adverse events.

Route	Hypotension (n = 37)	Bradycardia (n = 36)	Respiratory depression (n = 33)	Oxygen desaturation (n = 8)	Naloxone use (n = 75)
IV*	33	26	31	7	63
Oral	3	7	2	1	9
Epidural/PCA**	1	3	0	0	3
*p* value	0.2	0.4	0.02	0.4	0.3

*Intravenous.

**Patient-controlled analgesia.

Cardiovascular and respiratory adverse events occurred significantly more often in subjects who received IV hydromorphone at doses ranging between 0.2 and 0.3 mg every 4 h (*p* < 0.05).

## Discussion

The results showed that hydromorphone can cause serious cardiovascular and respiratory adverse events. A plausible explanation of this finding could be the lack of knowledge about the doses of hydromorphone that are equivalent to the doses of other opioids and the difference in potency between oral and IV hydromorphone. Additionally, the sensitivity of the Saudi population to small doses of hydromorphone could have led to this finding. However, our study has several limitations. This study was a retrospective cohort study with a small number of patients. In addition, there was no control arm receiving other opioids.

Globally, there have been several case reports of patients who died due to respiratory depression after receiving hydromorphone^16^. In addition, hydromorphone can cause hypotension, including orthostatic hypotension and syncope, which could be associated with histamine release. Hydromorphone has been found to be associated with vagally mediated sinus pauses, leading to a significant decrease in the heart rate of a patient with no known cardiac conduction disease^17^.

In conclusion, the prescription of hydromorphone should be made by a multidisciplinary team consisting of the primary specialist physician, a pain management specialist, a pharmacist, and well-trained nurses. All medical professionals should agree on the dosage, dosing frequency, and duration of the hydromorphone prescription. They should be able to recognize, monitor and manage any potential adverse effects of hydromorphone.

## Data Availability

The dataset used for the study is available without patient identifiers from the corresponding author on reasonable request.

## Abbreviations

IV: Intravenous route
PCA: Patient-controlled analgesia
HR: Heart rate
SBP: Systolic blood pressure
MAP: Mean arterial pressure
RR: Respiration rate
SpO_2_: Oxygen saturation
VRS: Verbal rating scale
